# Supplementary material for: Streamlined extraction of nucleic acids and metabolites from low- and high-biomass samples using isopropanol and matrix tubes
Source: Microbiol Spectr. 2025 Oct 10;13(11):e01912-25. doi: 10.1128/spectrum.01912-25 (PMC12584695; doi:10.1128/spectrum.01912-25)
Supplement: Supplemental figures and tables — Figures S1 to S7 and Tables S1 to S3. [file spectrum.01912-25-s0001.docx]

***Supplementary Material***

***for***

**Streamlined Extraction of Nucleic Acids and Metabolites from Low- and High-Biomass Samples Using Isopropanol and Matrix Tubes**

Caitriona Brennan*, Justin P. Shaffer*, Pedro Belda-Ferre, Ipsita Mohanty, Yuhan Weng, Kalen Cantrell, Gail Ackermann, Celeste Allaband, MacKenzie Bryant, Sawyer Farmer, Antonio González, Daniel McDonald, Cameron Martino, Michael J. Meehan, Gibraan Rahman, Rodolfo A. Salido, Tara Schwartz, Se Jin Song, Caitlin Tribelhorn, Helena M. Tubb, Pieter C. Dorrestein, Rob Knight

*These authors contributed equally


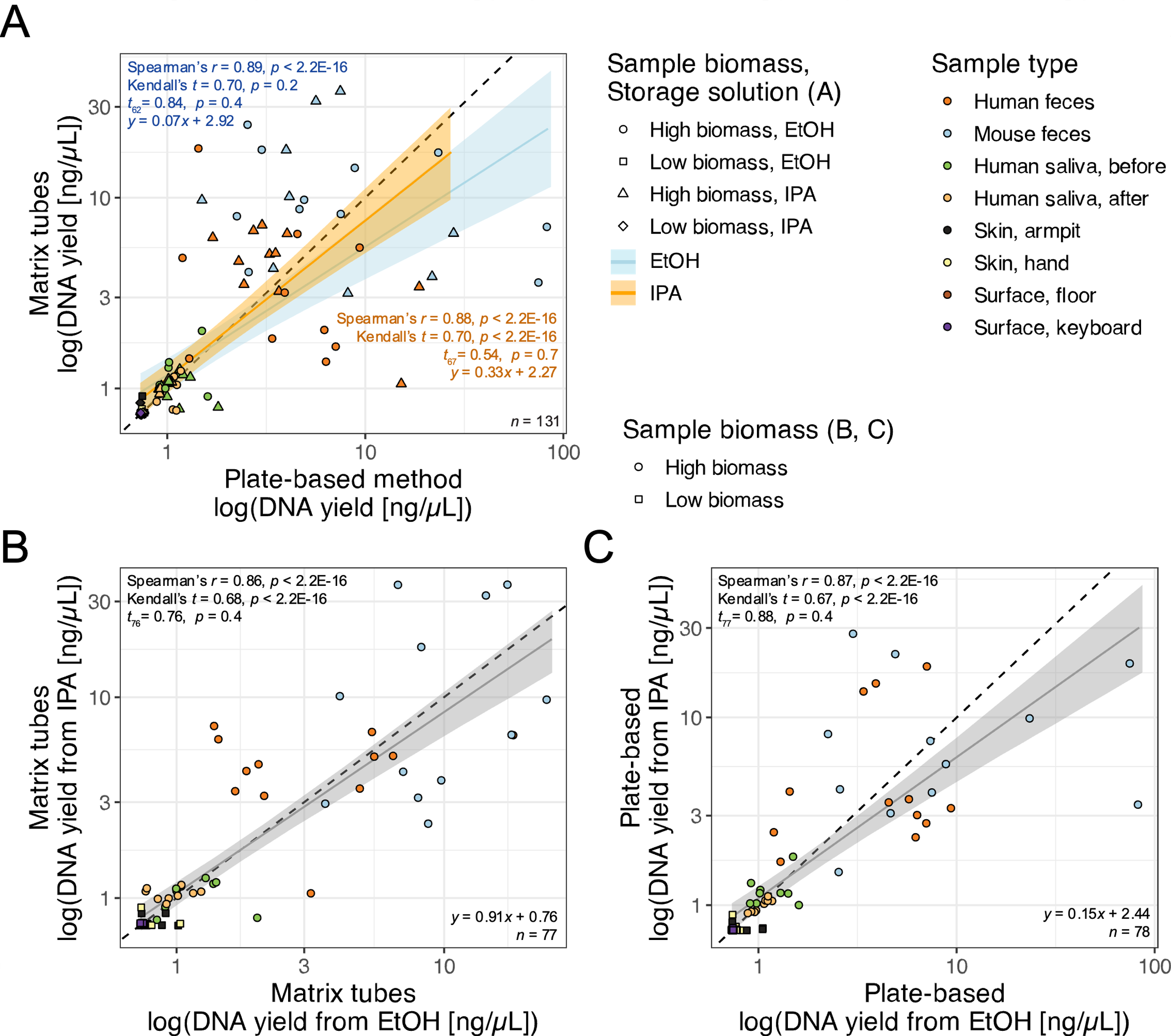


**Figure S1. DNA yield comparisons between extraction protocols and storage solutions within each protocol, for the same samples. (A)** The matrix method vs. the plate-based method. **(B)** Ethanol (EtOH) as a storage solution vs. isopropanol (IPA), for the Matrix method and **(C)** the plate-based method. For each panel, and for each storage solution in (A), results from correlation analyses are shown, as well as the equation for the linear model that best fits the data. For each storage solution in our comparison of extraction protocols in (A), we also include results from a one-sided, paired *t*-test (i.e., we expected more DNA from the plate-based method - see *Materials and Methods*). For comparison of storage solutions in (B) and (C), results from a two-sided, paired *t*-test are also shown. Each shaded area is a 95% confidence interval for the linear model shown, and dotted lines represent *y* = *x*.


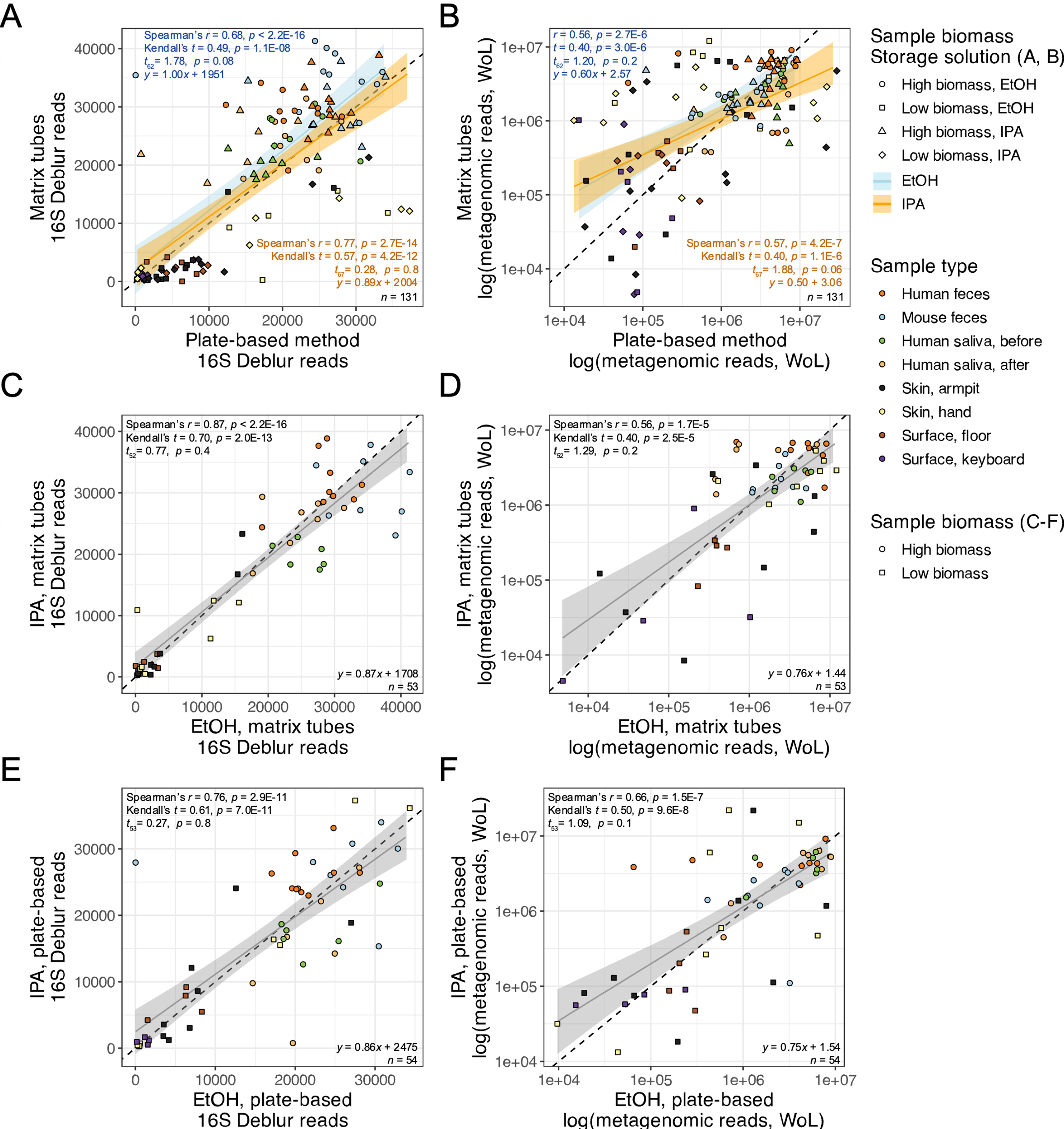


**Figure S2**. **Sequenced read count comparisons between extraction protocols and storage solutions within each protocol, for the same samples.** The matrix method vs. the plate-based method for **(A)** 16S data and **(B)** shotgun metagenomics data. Samples stored in ethanol (EtOH) vs. isopropanol (IPA) extracted with the matrix method for **(C)** 16S data and **(D)** shotgun metagenomics data. Samples stored in EtOH vs. IPA extracted with the plate-based method for **(E)** 16S data and **(F)** shotgun metagenomics data. Read counts for 16S data are Deblur reads. For each panel, results from correlation analyses and a two-sided paired *t*-test are shown, as well as the equation for the linear model that best fits the data. Shaded areas are 95% confidence intervals for the linear models shown, and dotted lines represent *y* = *x*.

**
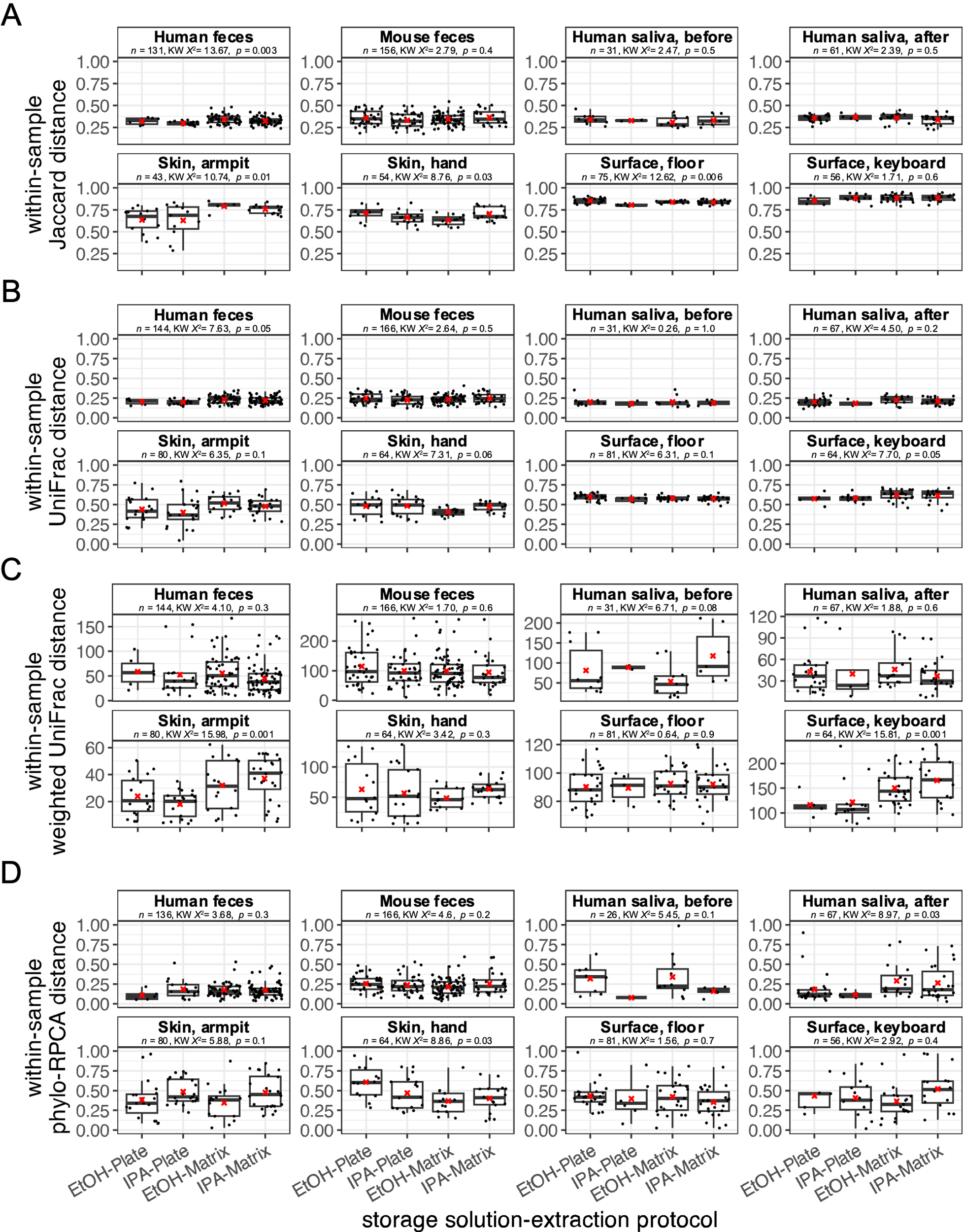
**

**Figure S3. Within-sample variation in microbial community composition across extraction protocols, for 16S data.** Microbial community beta-diversity among replicate extractions of the same source sample was estimated using (A) Jaccard distance, (B) unweighted UniFrac distance, (C) weighted UniFrac distance, and (D) phylogenetic RPCA distance. For each sample type, results from a Kruskal-Wallis test for differences among groups are shown. Data were normalized as in Table 2.

**
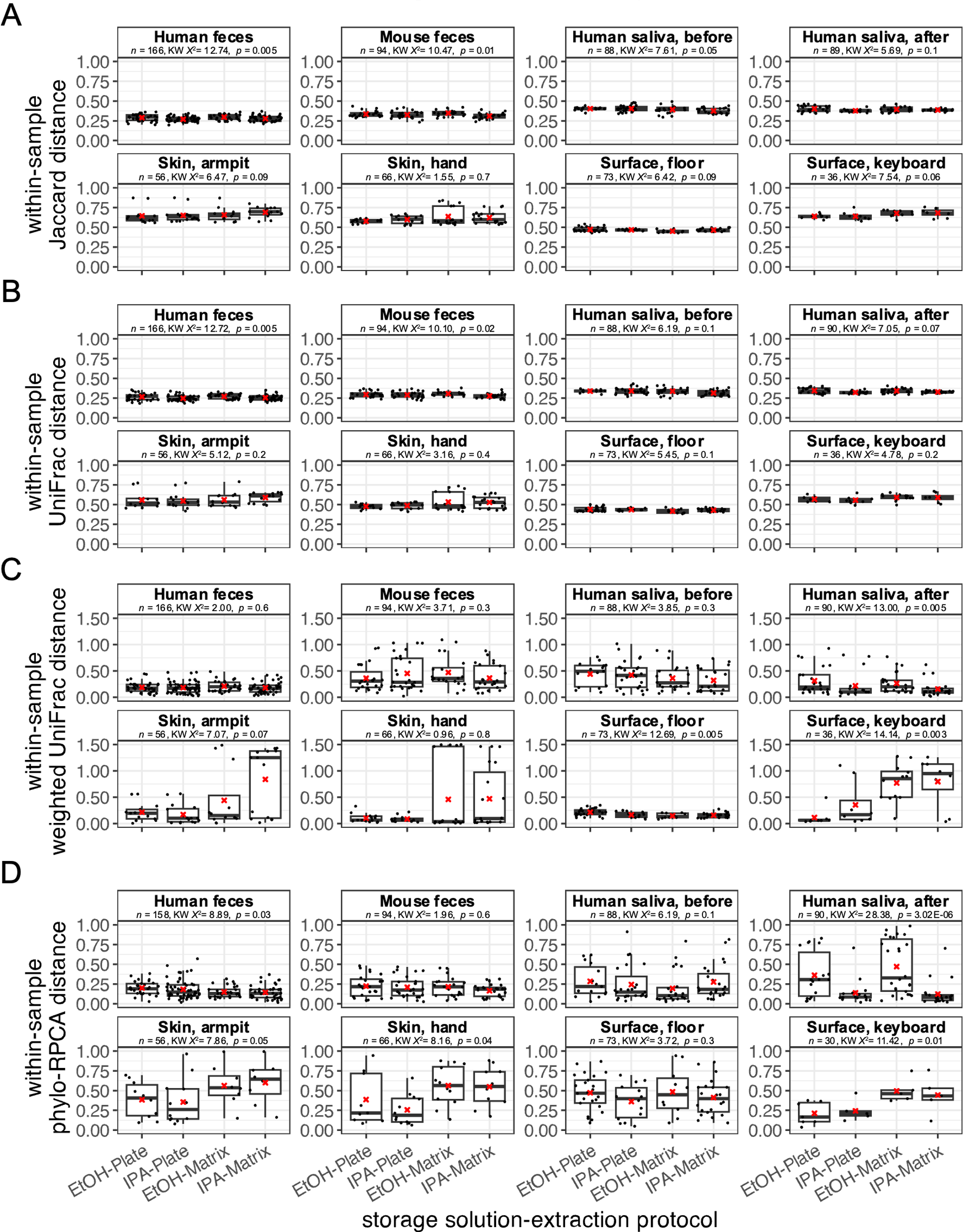
**

**Figure S4. Within-sample variation in microbial community composition across extraction protocols, for metagenomic data.** Microbial community beta-diversity among replicate extractions of the same source sample was estimated using (A) Jaccard distance, (B) unweighted UniFrac distance, (C) weighted UniFrac distance, and (D) phylogenetic RPCA distance. For each sample type, results from a Kruskal-Wallis test for differences among groups are shown. Data were normalized as in Table 2.


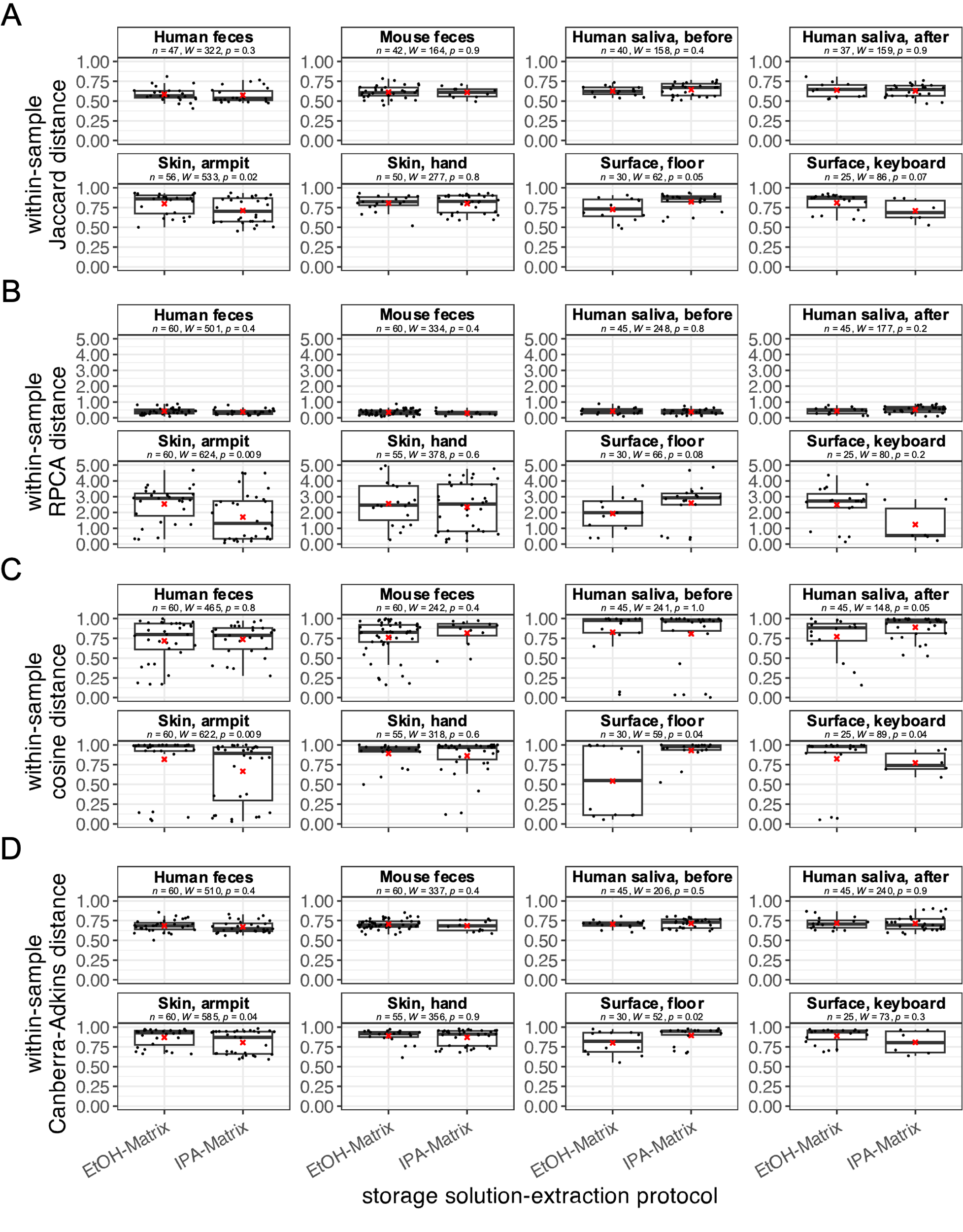


**Figure S5. Within-sample variation in metabolite composition across extraction protocols, for metabolomic data.** Metabolite beta-diversity among replicate extractions of the same source sample was estimated using (A) Jaccard distance, (B) RPCA distance, (C) cosine distance, and (D) Canberra-Adkins distance. Data were normalized as for table 2.

**
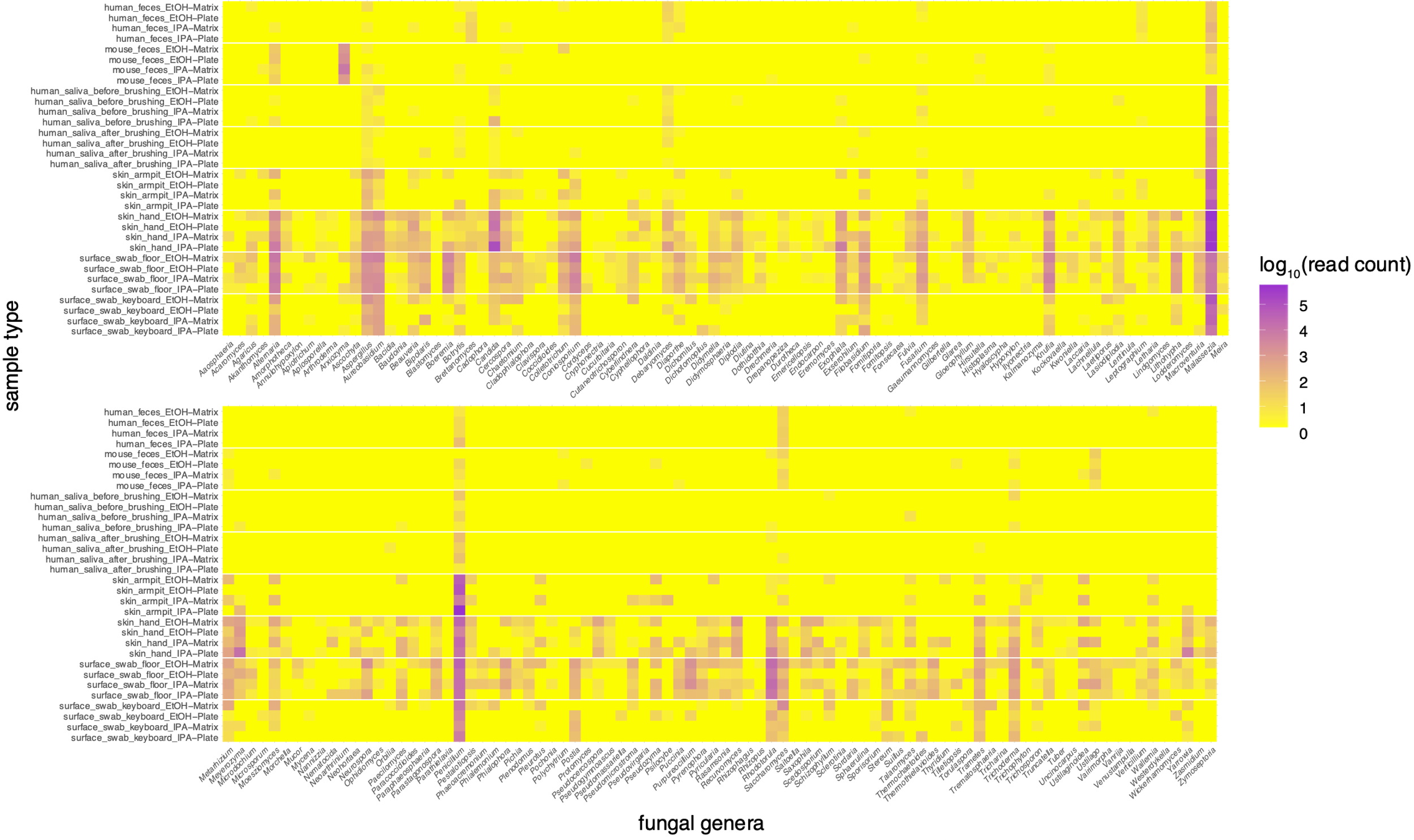
**

**Figure S6.** Heatmap of fungal genera represented in the analysis of taxon bias (i.e., UpSet plots) for Fungi (Figs. 6, 7). Fungal genera are listed in alphabetical order and the table is wrapped for visual clarity. Colors represent read counts (log transformed), where cooler/darker colors indicate higher counts.

**
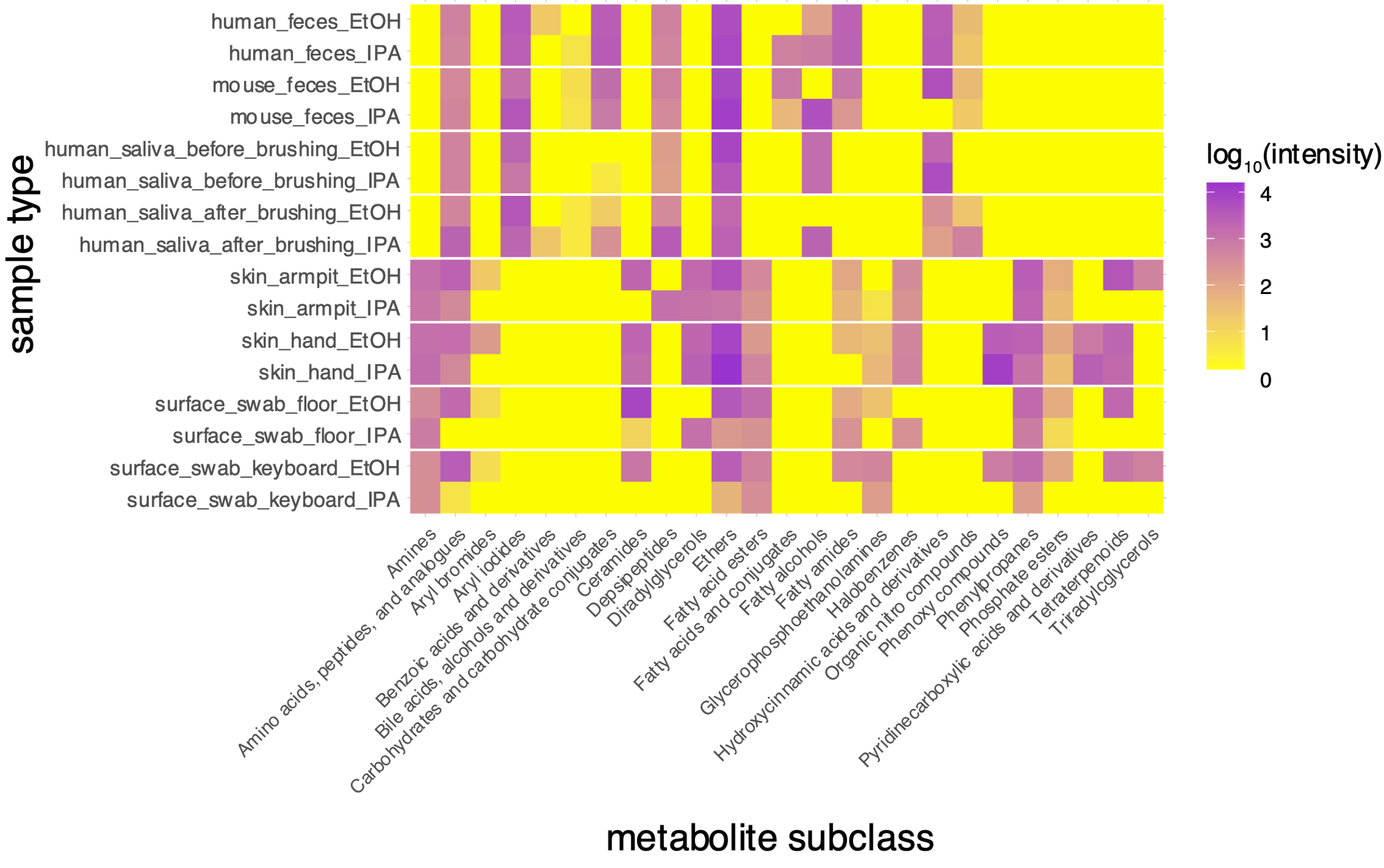
**

**Figure S7.** Heatmap of metabolite subclasses to complement our analysis of bias (i.e., UpSet plot) for metabolites (Fig. 8). Metabolite subclasses are listed in alphabetical order. Colors represent intensities (log transformed), where cooler/darker colors indicate higher values.

**Table S1. Results of dispersion analysis (PERMDISP) and Permutational Multivariate Analysis of Variance (PERMANOVA) for 16S data.** For each sample type, the effects of host subject, storage solution (i.e., ethanol vs. isopropanol), and extraction protocol (i.e., the plate-based- vs. the matrix method) are shown for five distance metrics. High- and low-biomass samples were rarefied to 20,636 and 277 quality-filtered reads per sample (or had samples with fewer than 20,636 and 277 reads excluded, for phylo-RPCA), respectively. Rarefaction depths were selected to maintain at least 75% of samples for each sample type. Each test included 999 permutations. The *adonis* framework was used for PERMANOVA to allow assessment of interaction terms.

|  |  |  |  | PERMDISP | | | | PERMANOVA (*adonis*) | | | |
| --- | --- | --- | --- | --- | --- | --- | --- | --- | --- | --- | --- |
| biomass | sample type | distance metric | factor | *n* | *k* | *F* | *p*-value | *df* | *F* | *R*2 | *p*-value |
| high | stool, human | Jaccard | host subject | 36 | 4 | 5.84 | **0.001** | 3 | 53.65 | 0.83 | **0.001** |
|  |  |  | storage solution | 36 | 2 | 0.22 | 0.5 | 1 | 1.13 | 0.007 | 0.4 |
|  |  |  | extraction protocol | 36 | 2 | 0.25 | 0.4 | 1 | 1.26 | 0.007 | 0.3 |
|  |  |  | storage solution x extraction protocol | not applicable | | | | 1 | 1.30 | 0.15 | 0.3 |
|  |  | RPCA | host subject | 36 | 4 | 0.13 | 0.9 | 3 | 36.69 | 0.78 | **0.001** |
|  |  |  | storage solution | 36 | 2 | 0.002 | 1.0 | 1 | 0.40 | 0.003 | 0.7 |
|  |  |  | extraction protocol | 36 | 2 | 0.25 | 0.6 | 1 | 0.59 | 0.004 | 0.6 |
|  |  |  | storage solution x extraction protocol | not applicable | | | | 1 | 1.63 | 0.01 | 0.2 |
|  |  | UniFrac | host subject | 36 | 4 | 8.88 | **0.001** | 3 | 61.17 | 0.85 | **0.001** |
|  |  |  | storage solution | 36 | 2 | 0.79 | 0.2 | 1 | 1.35 | 0.006 | 0.2 |
|  |  |  | extraction protocol | 36 | 2 | 0.32 | 0.4 | 1 | 1.58 | 0.007 | 0.2 |
|  |  |  | storage solution x extraction protocol | not applicable | | | | 1 | 1.38 | 0.006 | 0.2 |
|  |  | weighted UniFrac | host subject | 36 | 4 | 3.55 | **0.002** | 3 | 141.8 | 0.93 | **0.001** |
|  |  |  | storage solution | 36 | 2 | 0.09 | 0.8 | 1 | 2.08 | 0.005 | 0.1 |
|  |  |  | extraction protocol | 36 | 2 | 0.68 | 0.4 | 1 | 1.77 | 0.004 | 0.2 |
|  |  |  | storage solution x extraction protocol | not applicable | | | | 1 | 0.59 | 0.001 | 0.6 |
|  |  | phylo-RPCA | host subject | 36 | 4 | 1.47 | **0.05** | 3 | 95.75 | 0.89 | **0.001** |
|  |  |  | storage solution | 36 | 2 | 1.28 | 0.1 | 1 | 1.95 | 0.006 | 0.2 |
|  |  |  | extraction protocol | 36 | 2 | 1.93 | 0.06 | 1 | 0.34 | 0.001 | 0.7 |
|  |  |  | storage solution x extraction protocol | not applicable | | | | 1 | 3.43 | 0.01 | 0.06 |
|  | stool, mouse | Jaccard | host subject | 38 | 4 | 1.98 | **0.02** | 3 | 34.39 | 0.75 | **0.001** |
|  |  |  | storage solution | 38 | 2 | 2.13 | **0.03** | 1 | 1.25 | 0.01 | 0.3 |
|  |  |  | extraction protocol | 38 | 2 | 0.27 | 0.5 | 1 | 1.39 | 0.01 | 0.2 |
|  |  |  | storage solution x extraction protocol | not applicable | | | | 1 | 0.97 | 0.23 | 0.4 |
|  |  | RPCA | host subject | 38 | 4 | 0.41 | 0.6 | 3 | 50.19 | 0.82 | **0.001** |
|  |  |  | storage solution | 38 | 2 | 0.27 | 0.6 | 1 | 1.08 | 0.006 | 0.3 |
|  |  |  | extraction protocol | 38 | 2 | 0.20 | 0.6 | 1 | 1.28 | 0.007 | 0.3 |
|  |  |  | storage solution x extraction protocol | not applicable | | | | 1 | 0.60 | 0.003 | 0.5 |
|  |  | UniFrac | host subject | 38 | 4 | 1.36 | **0.05** | 3 | 40.26 | 0.77 | **0.001** |
|  |  |  | storage solution | 38 | 2 | 1.78 | 0.06 | 1 | 1.29 | 0.008 | 0.2 |
|  |  |  | extraction protocol | 38 | 2 | 1.03 | 0.2 | 1 | 2.20 | 0.01 | 0.1 |
|  |  |  | storage solution x extraction protocol | not applicable | | | | 1 | 0.93 | 0.006 | 0.4 |
|  |  | weighted UniFrac | host subject | 38 | 4 | 0.39 | 0.6 | 3 | 24.69 | 0.65 | **0.001** |
|  |  |  | storage solution | 38 | 2 | 0.06 | 0.8 | 1 | 0.19 | 0.002 | 0.9 |
|  |  |  | extraction protocol | 38 | 2 | 2.03 | 0.1 | 1 | 7.50 | 0.07 | **0.004** |
|  |  |  | storage solution x extraction protocol | not applicable | | | | 1 | 0.75 | 0.007 | 0.5 |
|  |  | phylo-RPCA | host subject | 38 | 4 | 0.55 | 0.6 | 3 | 15.58 | 0.56 | **0.001** |
|  |  |  | storage solution | 38 | 2 | 0.02 | 0.9 | 1 | 0.39 | 0.01 | 0.7 |
|  |  |  | extraction protocol | 38 | 2 | 1.11 | 0.3 | 1 | 5.34 | 0.06 | **0.01** |
|  |  |  | storage solution x extraction protocol | not applicable | | | | 1 | 0.53 | 0.006 | 0.6 |
|  | saliva, before brushing | Jaccard | host subject | 15 | 3 | 1.55 | **0.04** | 2 | 17.06 | 0.73 | **0.001** |
|  |  |  | storage solution | 15 | 2 | 0.70 | 0.4 | 1 | 1.70 | 0.04 | 0.1 |
|  |  |  | extraction protocol | 15 | 2 | 0.30 | 0.5 | 1 | 1.28 | 0.03 | 0.3 |
|  |  |  | storage solution x extraction protocol | not applicable | | | | 1 | 0.89 | 0.02 | 0.5 |
|  |  | RPCA | host subject | 15 | 3 | 0.32 | 0.7 | 2 | 7.40 | 0.61 | **0.001** |
|  |  |  | storage solution | 15 | 2 | 0.20 | 0.7 | 1 | 0.29 | 0.01 | 0.8 |
|  |  |  | extraction protocol | 15 | 2 | 1.86 | 0.1 | 1 | 0.07 | 0.003 | 0.9 |
|  |  |  | storage solution x extraction protocol | not applicable | | | | 1 | 0.1 | 0.004 | 0.9 |
|  |  | UniFrac | host subject | 15 | 3 | 0.50 | 0.5 | 2 | 14.22 | 0.68 | **0.001** |
|  |  |  | storage solution | 15 | 2 | 1.35 | 0.3 | 1 | 1.55 | 0.04 | 0.2 |
|  |  |  | extraction protocol | 15 | 2 | 0.0005 | 1.0 | 1 | 2.10 | 0.05 | 0.1 |
|  |  |  | storage solution x extraction protocol | not applicable | | | | 1 | 0.46 | 0.01 | 0.9 |
|  |  | weighted UniFrac | host subject | 15 | 3 | 0.35 | 0.6 | 2 | 20.74 | 0.58 | **0.001** |
|  |  |  | storage solution | 15 | 2 | 0.03 | 0.9 | 1 | 17.67 | 0.25 | **0.002** |
|  |  |  | extraction protocol | 15 | 2 | 1.45 | 0.2 | 1 | 1.86 | 0.03 | 0.2 |
|  |  |  | storage solution x extraction protocol | not applicable | | | | 1 | 1.51 | 0.02 | 0.2 |
|  |  | phylo-RPCA | host subject | 15 | 3 | 3.29 | 0.07 | 2 | 5.13 | 0.39 | **0.001** |
|  |  |  | storage solution | 15 | 2 | 0.12 | 0.8 | 1 | 0.52 | 0.02 | 0.7 |
|  |  |  | extraction protocol | 15 | 2 | 0.32 | 0.5 | 1 | 3.93 | 0.15 | **0.02** |
|  |  |  | storage solution x extraction protocol | not applicable | | | | 1 | 2.31 | 0.09 | 0.2 |
|  | saliva, after brushing | Jaccard | host subject | 19 | 3 | 18.81 | **0.02** | 2 | 16.86 | 0.67 | **0.001** |
|  |  |  | storage solution | 19 | 2 | 0.53 | 0.3 | 1 | 1.43 | 0.03 | 0.2 |
|  |  |  | extraction protocol | 19 | 2 | 1.91 | 0.09 | 1 | 1.05 | 0.02 | 0.4 |
|  |  |  | storage solution x extraction protocol | not applicable | | | | 1 | 0.88 | 0.26 | 0.4 |
|  |  | RPCA | host subject | 19 | 3 | 0.57 | 0.6 | 2 | 16.74 | 0.68 | **0.001** |
|  |  |  | storage solution | 19 | 2 | 1.08 | 0.2 | 1 | 0.85 | 0.02 | 0.4 |
|  |  |  | extraction protocol | 19 | 2 | 0.39 | 0.5 | 1 | 0.26 | 0.005 | 0.7 |
|  |  |  | storage solution x extraction protocol | not applicable | | | | 1 | 1.89 | 0.04 | 0.2 |
|  |  | UniFrac | host subject | 19 | 3 | 3.88 | 0.1 | 2 | 14.30 | 0.63 | **0.001** |
|  |  |  | storage solution | 19 | 2 | 0.91 | 0.3 | 1 | 1.69 | 0.04 | 0.1 |
|  |  |  | extraction protocol | 19 | 2 | 1.94 | 0.1 | 1 | 0.64 | 0.01 | 0.7 |
|  |  |  | storage solution x extraction protocol | not applicable | | | | 1 | 1.25 | 0.03 | 0.2 |
|  |  | weighted UniFrac | host subject | 19 | 3 | 0.08 | 0.9 | 2 | 76.67 | 0.87 | **0.001** |
|  |  |  | storage solution | 19 | 2 | 0.20 | 0.5 | 1 | 3.45 | 0.02 | 0.07 |
|  |  |  | extraction protocol | 19 | 2 | 1.14 | 0.2 | 1 | 5.83 | 0.03 | **0.01** |
|  |  |  | storage solution x extraction protocol | not applicable | | | | 1 | 1.01 | 0.006 | 0.3 |
|  |  | phylo-RPCA | host subject | 19 | 3 | 19.76 | **0.001** | 2 | 9.06 | 0.52 | **0.003** |
|  |  |  | storage solution | 19 | 2 | 1.48 | 0.2 | 1 | 2.36 | 0.07 | 0.1 |
|  |  |  | extraction protocol | 19 | 2 | 0.31 | 0.6 | 1 | 0.06 | 0.002 | 0.9 |
|  |  |  | storage solution x extraction protocol | not applicable | | | | 1 | 1.43 | 0.04 | 0.2 |
| low | skin, armpit | Jaccard | host subject | 40 | 4 | 1.29 | 0.1 | 3 | 4.49 | 0.25 | **0.001** |
|  |  |  | storage solution | 40 | 2 | 0.31 | 0.6 | 1 | 0.75 | 0.01 | 0.9 |
|  |  |  | extraction protocol | 40 | 2 | 3.42 | **0.05** | 1 | 4.94 | 0.09 | **0.001** |
|  |  |  | storage solution x extraction protocol | not applicable | | | | 1 | 0.97 | 0.02 | 0.5 |
|  |  | RPCA | host subject | 40 | 4 | 1.05 | 0.4 | 3 | 14.12 | 0.54 | **0.001** |
|  |  |  | storage solution | 40 | 2 | 0.0005 | 1.0 | 1 | 1.07 | 0.01 | 0.4 |
|  |  |  | extraction protocol | 40 | 2 | 1.40 | 0.3 | 1 | 2.06 | 0.03 | 0.1 |
|  |  |  | storage solution x extraction protocol | not applicable | | | | 1 | 0.30 | 0.004 | 0.8 |
|  |  | UniFrac | host subject | 40 | 4 | 0.39 | 0.6 | 3 | 6.82 | 0.34 | **0.001** |
|  |  |  | storage solution | 40 | 2 | 0.003 | 1.0 | 1 | 0.50 | 0.009 | 0.9 |
|  |  |  | extraction protocol | 40 | 2 | 0.58 | 0.4 | 1 | 6.30 | 0.10 | **0.001** |
|  |  |  | storage solution x extraction protocol | not applicable | | | | 1 | 0.65 | 0.01 | 0.8 |
|  |  | weighted UniFrac | host subject | 40 | 4 | 7.58 | **0.003** | 3 | 23.41 | 0.63 | **0.001** |
|  |  |  | storage solution | 40 | 2 | 0.42 | 0.5 | 1 | 0.82 | 0.007 | 0.5 |
|  |  |  | extraction protocol | 40 | 2 | 0.52 | 0.5 | 1 | 6.93 | 0.06 | **0.001** |
|  |  |  | storage solution x extraction protocol | not applicable | | | | 1 | 1.05 | 0.009 | 0.4 |
|  |  | phylo-RPCA | host subject | 40 | 4 | 0.24 | 0.8 | 3 | 41.87 | 0.76 | **0.001** |
|  |  |  | storage solution | 40 | 2 | 1.83 | 0.2 | 1 | 0.81 | 0.005 | 0.4 |
|  |  |  | extraction protocol | 40 | 2 | 1.32 | 0.2 | 1 | 6.42 | 0.04 | **0.009** |
|  |  |  | storage solution x extraction protocol | not applicable | | | | 1 | 0.31 | 0.002 | 0.7 |
|  | skin, hand | Jaccard | host subject | 36 | 4 | 31.20 | **0.001** | 3 | 5.42 | 0.32 | **0.001** |
|  |  |  | storage solution | 36 | 2 | 0.81 | 0.3 | 1 | 0.95 | 0.02 | 0.5 |
|  |  |  | extraction protocol | 36 | 2 | 9.76 | **0.008** | 1 | 3.54 | 0.07 | **0.001** |
|  |  |  | storage solution x extraction protocol | not applicable | | | | 1 | 0.88 | 0.02 | 0.6 |
|  |  | RPCA | host subject | 36 | 4 | 2.17 | 0.07 | 3 | 106.66 | 0.89 | **0.001** |
|  |  |  | storage solution | 36 | 2 | 0.83 | 0.3 | 1 | 0.10 | 0.0003 | 0.9 |
|  |  |  | extraction protocol | 36 | 2 | 3.29 | 0.08 | 1 | 9.02 | 0.03 | **0.001** |
|  |  |  | storage solution x extraction protocol | not applicable | | | | 1 | 0.22 | 0.0006 | 0.8 |
|  |  | UniFrac | host subject | 36 | 4 | 29.10 | 0.001 | 3 | 5.24 | 0.30 | **0.001** |
|  |  |  | storage solution | 36 | 2 | 0.63 | 0.4 | 1 | 0.81 | 0.02 | 0.7 |
|  |  |  | extraction protocol | 36 | 2 | 8.30 | 0.02 | 1 | 5.56 | 0.11 | **0.001** |
|  |  |  | storage solution x extraction protocol | not applicable | | | | 1 | 0.74 | 0.01 | 0.7 |
|  |  | weighted UniFrac | host subject | 36 | 4 | 1.73 | 0.1 | 3 | 16.76 | 0.58 | **0.001** |
|  |  |  | storage solution | 36 | 2 | 0.27 | 0.6 | 1 | 0.31 | 0.004 | 0.9 |
|  |  |  | extraction protocol | 36 | 2 | 3.23 | 0.09 | 1 | 7.23 | 0.08 | **0.001** |
|  |  |  | storage solution x extraction protocol | not applicable | | | | 1 | 0.31 | 0.004 | 0.9 |
|  |  | phylo-RPCA | host subject | 36 | 4 | 2.00 | **0.02** | 3 | 65.02 | 0.84 | **0.001** |
|  |  |  | storage solution | 36 | 2 | 0.05 | 0.7 | 1 | 0.34 | 0.001 | 0.6 |
|  |  |  | extraction protocol | 36 | 2 | 0.56 | 0.2 | 1 | 8.38 | 0.36 | **0.007** |
|  |  |  | storage solution x extraction protocol | not applicable | | | | 1 | 0.26 | 0.001 | 0.7 |
|  | surface, floor tile | Jaccard | host subject | 19 | 2 | 2.68 | 0.1 | 1 | 1.52 | 0.08 | **0.001** |
|  |  |  | storage solution | 19 | 2 | 0.44 | 0.5 | 1 | 1.02 | 0.05 | 0.4 |
|  |  |  | extraction protocol | 19 | 2 | 0.36 | 0.5 | 1 | 1.23 | 0.07 | **0.004** |
|  |  |  | storage solution x extraction protocol | not applicable | | | | 1 | 0.97 | 0.05 | 0.7 |
|  |  | RPCA | host subject | 19 | 2 | 0.24 | 0.6 | 1 | 2.62 | 0.08 | 0.1 |
|  |  |  | storage solution | 19 | 2 | 0.0002 | 1.0 | 1 | 1.66 | 0.05 | 0.2 |
|  |  |  | extraction protocol | 19 | 2 | 1.16 | 0.2 | 1 | 13.84 | 0.42 | **0.001** |
|  |  |  | storage solution x extraction protocol | not applicable | | | | 1 | 0.80 | 0.02 | 0.4 |
|  |  | UniFrac | host subject | 19 | 2 | 0.41 | 0.5 | 1 | 1.47 | 0.08 | **0.001** |
|  |  |  | storage solution | 19 | 2 | 0.05 | 0.8 | 1 | 0.98 | 0.05 | 0.6 |
|  |  |  | extraction protocol | 19 | 2 | 1.05 | 0.3 | 1 | 1.41 | 0.08 | **0.002** |
|  |  |  | storage solution x extraction protocol | not applicable | | | | 1 | 0.96 | 0.05 | 0.6 |
|  |  | weighted UniFrac | host subject | 19 | 2 | 0.21 | 0.6 | 1 | 3.89 | 0.18 | **0.001** |
|  |  |  | storage solution | 19 | 2 | 1.70 | 0.2 | 1 | 0.91 | 0.04 | 0.5 |
|  |  |  | extraction protocol | 19 | 2 | 0.07 | 0.8 | 1 | 2.14 | 0.10 | **0.01** |
|  |  |  | storage solution x extraction protocol | not applicable | | | | 1 | 0.76 | 0.04 | 0.7 |
|  |  | phylo-RPCA | host subject | 19 | 2 | 1.26 | 0.2 | 1 | 2.12 | 0.11 | 0.2 |
|  |  |  | storage solution | 19 | 2 | 0.43 | 0.4 | 1 | 0.009 | 0.005 | 1 |
|  |  |  | extraction protocol | 19 | 2 | 0.58 | 0.4 | 1 | 1.82 | 0.10 | 0.2 |
|  |  |  | storage solution x extraction protocol | not applicable | | | | 1 | 0.61 | 0.03 | 0.6 |
|  | surface, keyboard | Jaccard | host subject | 17 | 2 | 2.49 | 0.1 | 1 | 1.01 | 0.06 | 0.4 |
|  |  |  | storage solution | 17 | 2 | 0.30 | 0.5 | 1 | 0.98 | 0.06 | 0.6 |
|  |  |  | extraction protocol | 17 | 2 | 0.08 | 0.8 | 1 | 1.48 | 0.09 | **0.001** |
|  |  |  | storage solution x extraction protocol | not applicable | | | | 1 | 1.01 | 0.06 | 0.4 |
|  |  | RPCA | host subject | 17 | 2 | 0.13 | 0.7 | 1 | 1.70 | 0.08 | 0.2 |
|  |  |  | storage solution | 17 | 2 | 0.88 | 0.3 | 1 | 0.85 | 0.04 | 0.3 |
|  |  |  | extraction protocol | 17 | 2 | 0.10 | 0.8 | 1 | 6.25 | 0.30 | **0.01** |
|  |  |  | storage solution x extraction protocol | not applicable | | | | 1 | 0.11 | 0.005 | 0.9 |
|  |  | UniFrac | host subject | 17 | 2 | 0.94 | 0.3 | 1 | 1.25 | 0.07 | 0.1 |
|  |  |  | storage solution | 17 | 2 | 0.01 | 0.9 | 1 | 1.31 | 0.07 | 0.07 |
|  |  |  | extraction protocol | 17 | 2 | 0.005 | 0.9 | 1 | 1.85 | 0.10 | **0.001** |
|  |  |  | storage solution x extraction protocol | not applicable | | | | 1 | 1.23 | 0.07 | 0.1 |
|  |  | weighted UniFrac | host subject | 17 | 2 | 0.25 | 0.7 | 1 | 1.21 | 0.06 | 0.2 |
|  |  |  | storage solution | 17 | 2 | 2.33 | 0.1 | 1 | 1.16 | 0.06 | 0.3 |
|  |  |  | extraction protocol | 17 | 2 | 5.94 | **0.02** | 1 | 4.80 | 0.23 | **0.001** |
|  |  |  | storage solution x extraction protocol | not applicable | | | | 1 | 1.47 | 0.07 | 0.1 |
|  |  | phylo-RPCA | host subject | 17 | 2 | 7.77 | **0.01** | 1 | 0.61 | 0.03 | 0.6 |
|  |  |  | storage solution | 17 | 2 | 0.38 | 0.6 | 1 | 0.55 | 0.03 | 0.6 |
|  |  |  | extraction protocol | 17 | 2 | 2.03 | 0.2 | 1 | 7.48 | 0.36 | **0.003** |
|  |  |  | storage solution x extraction protocol | not applicable | | | | 1 | 0.24 | 0.01 | 0.8 |

**Table S2. Results of dispersion analysis (PERMDISP) and Permutational Multivariate Analysis of Variance (PERMANOVA) for shotgun metagenomic data.** For each sample type, the effects of host subject, storage solution (i.e., ethanol vs. isopropanol), and extraction protocol (i.e., the plate-based- vs. the matrix method) are shown for five distance metrics. High- and low-biomass samples were rarefied to 1,515,275 and 55,892 quality-filtered reads per sample (or had samples with fewer than 1,515,275 and 55,892 reads excluded, for phylo-RPCA), respectively. Rarefaction depths were selected to maintain at least 75% of samples for each sample type. Each test included 999 permutations. The *adonis* framework was used for PERMANOVA to allow assessment of interaction terms.

|  |  |  |  | PERMDISP | | | | PERMANOVA (*adonis*) | | | |
| --- | --- | --- | --- | --- | --- | --- | --- | --- | --- | --- | --- |
| biomass | sample type | distance metric | factor | *n* | *k* | *F* | *p*-value | *df* | *F* | *R*2 | *p*-value |
| high | stool, human | Jaccard | host subject | 38 | 4 | 14.80 | **0.001** | 3 | 16.40 | 0.58 | **0.001** |
|  |  |  | storage solution | 38 | 2 | 3.93 | **0.02** | 1 | 1.48 | 0.02 | 0.1 |
|  |  |  | extraction protocol | 38 | 2 | 0.11 | 0.7 | 1 | 1.63 | 0.02 | 0.1 |
|  |  |  | storage solution x extraction protocol | not applicable | | | | 1 | 1.03 | 0.01 | 0.4 |
|  |  | RPCA | host subject | 38 | 4 | 1.89 | **0.04** | 3 | 20.70 | 0.65 | **0.001** |
|  |  |  | storage solution | 38 | 2 | 0.06 | 0.8 | 1 | 1.06 | 0.01 | 0.3 |
|  |  |  | extraction protocol | 38 | 2 | 0.52 | 0.3 | 1 | 0.73 | 0.008 | 0.5 |
|  |  |  | storage solution x extraction protocol | not applicable | | | | 1 | 0.20 | 0.002 | 0.8 |
|  |  | UniFrac | host subject | 38 | 4 | 10.32 | **0.001** | 3 | 17.69 | 0.60 | **0.001** |
|  |  |  | storage solution | 38 | 2 | 3.07 | **0.04** | 1 | 1.43 | 0.02 | 0.2 |
|  |  |  | extraction protocol | 38 | 2 | 0.07 | 0.8 | 1 | 1.65 | 0.02 | 0.08 |
|  |  |  | storage solution x extraction protocol | not applicable | | | | 1 | 1.08 | 0.01 | 0.3 |
|  |  | weighted UniFrac | host subject | 38 | 4 | 2.49 | **0.05** | 3 | 111.38 | 0.90 | **0.001** |
|  |  |  | storage solution | 38 | 2 | 2.00E-06 | 1.0 | 1 | 0.89 | 0.002 | 0.4 |
|  |  |  | extraction protocol | 38 | 2 | 0.66 | 0.4 | 1 | 2.60 | 0.007 | 0.07 |
|  |  |  | storage solution x extraction protocol | not applicable | | | | 1 | 0.78 | 0.002 | 0.5 |
|  |  | phylo-RPCA | host subject | 38 | 4 | 1.83 | **0.05** | 3 | 20.51 | 0.63 | **0.001** |
|  |  |  | storage solution | 38 | 2 | 0.88 | 0.3 | 1 | 2.82 | 0.03 | 0.07 |
|  |  |  | extraction protocol | 38 | 2 | 0.02 | 0.9 | 1 | 1.43 | 0.01 | 0.2 |
|  |  |  | storage solution x extraction protocol | not applicable | | | | 1 | 0.61 | 0.006 | 0.5 |
|  | stool, mouse | Jaccard | host subject | 26 | 4 | 3.83 | 0.2 | 3 | 6.77 | 0.47 | **0.001** |
|  |  |  | storage solution | 26 | 2 | 0.27 | 0.6 | 1 | 1.01 | 0.02 | 0.3 |
|  |  |  | extraction protocol | 26 | 2 | 0.30 | 0.6 | 1 | 1.32 | 0.03 | 0.2 |
|  |  |  | storage solution x extraction protocol | not applicable | | | | 1 | 1.14 | 0.03 | 0.3 |
|  |  | RPCA | host subject | 26 | 4 | 1.88 | 0.1 | 3 | 17.98 | 0.71 | **0.001** |
|  |  |  | storage solution | 26 | 2 | 0.25 | 0.6 | 1 | 0.81 | 0.01 | 0.4 |
|  |  |  | extraction protocol | 26 | 2 | 0.30 | 0.5 | 1 | 0.41 | 0.005 | 0.6 |
|  |  |  | storage solution x extraction protocol | not applicable | | | | 1 | 1.46 | 0.02 | 0.2 |
|  |  | UniFrac | host subject | 26 | 4 | 3.14 | 0.3 | 3 | 6.64 | 0.47 | **0.001** |
|  |  |  | storage solution | 26 | 2 | 0.21 | 0.6 | 1 | 1.02 | 0.02 | 0.4 |
|  |  |  | extraction protocol | 26 | 2 | 0.83 | 0.3 | 1 | 1.32 | 0.03 | 0.2 |
|  |  |  | storage solution x extraction protocol | not applicable | | | | 1 | 1.10 | 0.03 | 0.3 |
|  |  | weighted UniFrac | host subject | 26 | 4 | 0.89 | 0.5 | 3 | 5.69 | 0.45 | **0.001** |
|  |  |  | storage solution | 26 | 2 | 0.06 | 0.8 | 1 | 0.31 | 0.008 | 0.9 |
|  |  |  | extraction protocol | 26 | 2 | 0.65 | 0.4 | 1 | 1.14 | 0.03 | 0.4 |
|  |  |  | storage solution x extraction protocol | not applicable | | | | 1 | 0.65 | 0.02 | 0.6 |
|  |  | phylo-RPCA | host subject | 26 | 4 | 1.35 | 0.2 | 3 | 26.15 | 0.78 | **0.001** |
|  |  |  | storage solution | 26 | 2 | 0.02 | 0.9 | 1 | 0.18 | 0.002 | 0.8 |
|  |  |  | extraction protocol | 26 | 2 | 0.28 | 0.5 | 1 | 0.48 | 0.005 | 0.5 |
|  |  |  | storage solution x extraction protocol | not applicable | | | | 1 | 2.51 | 0.02 | 0.1 |
|  | saliva, before brushing | Jaccard | host subject | 24 | 3 | 1.37 | 0.3 | 2 | 4.78 | 0.30 | **0.001** |
|  |  |  | storage solution | 24 | 2 | 0.49 | 0.5 | 1 | 2.43 | 0.08 | **0.002** |
|  |  |  | extraction protocol | 24 | 2 | 0.33 | 0.5 | 1 | 1.15 | 0.04 | 0.2 |
|  |  |  | storage solution x extraction protocol | not applicable | | | | 1 | 0.93 | 0.03 | 0.5 |
|  |  | RPCA | host subject | 24 | 3 | 1.03 | 0.3 | 2 | 15.92 | 0.59 | **0.001** |
|  |  |  | storage solution | 24 | 2 | 0.71 | 0.4 | 1 | 2.87 | 0.05 | 0.1 |
|  |  |  | extraction protocol | 24 | 2 | 0.004 | 1.0 | 1 | 0.57 | 0.01 | 0.6 |
|  |  |  | storage solution x extraction protocol | not applicable | | | | 1 | 1.05 | 0.02 | 0.4 |
|  |  | UniFrac | host subject | 24 | 3 | 1.11 | 0.3 | 2 | 5.00 | 0.31 | **0.001** |
|  |  |  | storage solution | 24 | 2 | 0.72 | 0.4 | 1 | 2.53 | 0.08 | **0.003** |
|  |  |  | extraction protocol | 24 | 2 | 0.06 | 0.8 | 1 | 1.17 | 0.04 | 0.2 |
|  |  |  | storage solution x extraction protocol | not applicable | | | | 1 | 0.91 | 0.03 | 0.6 |
|  |  | weighted UniFrac | host subject | 24 | 3 | 1.69 | 0.2 | 2 | 29.50 | 0.54 | **0.001** |
|  |  |  | storage solution | 24 | 2 | 1.16 | 0.2 | 1 | 31.85 | 0.29 | **0.001** |
|  |  |  | extraction protocol | 24 | 2 | 0.29 | 0.6 | 1 | 0.56 | 0.005 | 0.6 |
|  |  |  | storage solution x extraction protocol | not applicable | | | | 1 | 0.85 | 0.008 | 0.4 |
|  |  | phylo-RPCA | host subject | 24 | 3 | 1.79 | 0.2 | 2 | 10.15 | 0.51 | **0.001** |
|  |  |  | storage solution | 24 | 2 | 0.20 | 0.6 | 1 | 0.40 | 0.01 | 0.6 |
|  |  |  | extraction protocol | 24 | 2 | 0.01 | 0.9 | 1 | 0.60 | 0.02 | 0.5 |
|  |  |  | storage solution x extraction protocol | not applicable | | | | 1 | 0.86 | 0.02 | 0.4 |
|  | saliva, after brushing | Jaccard | host subject | 21 | 3 | 159.70 | **0.02** | 2 | 3.69 | 0.28 | **0.001** |
|  |  |  | storage solution | 21 | 2 | 0.05 | 0.8 | 1 | 1.45 | 0.06 | 0.07 |
|  |  |  | extraction protocol | 21 | 2 | 0.01 | 0.9 | 1 | 1.21 | 0.05 | 0.2 |
|  |  |  | storage solution x extraction protocol | not applicable | | | | 1 | 0.92 | 0.04 | 0.5 |
|  |  | RPCA | host subject | 21 | 3 | 4.05 | 0.06 | 2 | 9.78 | 0.51 | **0.001** |
|  |  |  | storage solution | 21 | 2 | 9.36 | **0.03** | 1 | 3.73 | 0.10 | 0.06 |
|  |  |  | extraction protocol | 21 | 2 | 0.02 | 0.9 | 1 | 0.11 | 0.003 | 0.9 |
|  |  |  | storage solution x extraction protocol | not applicable | | | | 1 | 0.08 | 0.002 | 0.9 |
|  |  | UniFrac | host subject | 21 | 3 | 129.70 | **0.003** | 2 | 3.81 | 0.29 | **0.001** |
|  |  |  | storage solution | 21 | 2 | 0.04 | 0.8 | 1 | 1.48 | 0.06 | 0.07 |
|  |  |  | extraction protocol | 21 | 2 | 0.0001 | 1.0 | 1 | 1.24 | 0.05 | 0.2 |
|  |  |  | storage solution x extraction protocol | not applicable | | | | 1 | 0.91 | 0.03 | 0.5 |
|  |  | weighted UniFrac | host subject | 21 | 3 | 4.16 | **0.02** | 2 | 19.83 | 0.65 | **0.001** |
|  |  |  | storage solution | 21 | 2 | 1.04 | 0.2 | 1 | 4.87 | 0.08 | **0.02** |
|  |  |  | extraction protocol | 21 | 2 | 0.03 | 0.9 | 1 | 0.90 | 0.01 | 0.4 |
|  |  |  | storage solution x extraction protocol | not applicable | | | | 1 | 0.79 | 0.01 | 0.4 |
|  |  | phylo-RPCA | host subject | 21 | 3 | 3.61 | **0.04** | 2 | 10.35 | 0.52 | **0.002** |
|  |  |  | storage solution | 21 | 2 | 10.62 | **0.03** | 1 | 3.66 | 0.09 | 0.07 |
|  |  |  | extraction protocol | 21 | 2 | 0.01 | 0.9 | 1 | 0.27 | 0.007 | 0.7 |
|  |  |  | storage solution x extraction protocol | not applicable | | | | 1 | 0.08 | 0.002 | 0.9 |
| low | skin, armpit | Jaccard | host subject | 33 | 4 | 1.27 | 0.4 | 3 | 2.91 | 0.22 | **0.001** |
|  |  |  | storage solution | 33 | 2 | 0.0007 | 1.0 | 1 | 0.97 | 0.02 | 0.5 |
|  |  |  | extraction protocol | 33 | 2 | 0.0004 | 1.0 | 1 | 3.05 | 0.08 | **0.001** |
|  |  |  | storage solution x extraction protocol | not applicable | | | | 1 | 0.83 | 0.02 | 0.8 |
|  |  | RPCA | host subject | 33 | 4 | 0.45 | 0.7 | 3 | 4.13 | 0.28 | **0.004** |
|  |  |  | storage solution | 33 | 2 | 0.06 | 0.8 | 1 | 0.48 | 0.01 | 0.6 |
|  |  |  | extraction protocol | 33 | 2 | 4.74 | **0.03** | 1 | 2.84 | 0.07 | 0.1 |
|  |  |  | storage solution x extraction protocol | not applicable | | | | 1 | 1.97 | 0.05 | 0.1 |
|  |  | UniFrac | host subject | 33 | 4 | 0.38 | 0.8 | 3 | 3.14 | 0.23 | **0.001** |
|  |  |  | storage solution | 33 | 2 | 0.01 | 0.9 | 1 | 1.01 | 0.03 | 0.4 |
|  |  |  | extraction protocol | 33 | 2 | 0.0005 | 1.0 | 1 | 2.90 | 0.07 | **0.001** |
|  |  |  | storage solution x extraction protocol | not applicable | | | | 1 | 0.78 | 0.02 | 0.8 |
|  |  | weighted UniFrac | host subject | 33 | 4 | 0.32 | 0.8 | 3 | 5.14 | 0.32 | **0.001** |
|  |  |  | storage solution | 33 | 2 | 0.25 | 0.6 | 1 | 0.64 | 0.01 | 0.6 |
|  |  |  | extraction protocol | 33 | 2 | 5.85 | **0.03** | 1 | 4.95 | 0.10 | **0.007** |
|  |  |  | storage solution x extraction protocol | not applicable | | | | 1 | 0.70 | 0.01 | 0.5 |
|  |  | phylo-RPCA | host subject | 33 | 4 | 1.64 | 0.1 | 3 | 7.17 | 0.41 | **0.001** |
|  |  |  | storage solution | 33 | 2 | 0.17 | 0.7 | 1 | 2.68 | 0.05 | 0.08 |
|  |  |  | extraction protocol | 33 | 2 | 1.28 | 0.2 | 1 | 0.06 | 0.001 | 0.9 |
|  |  |  | storage solution x extraction protocol | not applicable | | | | 1 | 2.32 | 0.04 | 0.1 |
|  | skin, hand | Jaccard | host subject | 34 | 4 | 3.59 | 0.1 | 3 | 4.27 | 0.29 | **0.001** |
|  |  |  | storage solution | 34 | 2 | 0.29 | 0.6 | 1 | 0.95 | 0.02 | 0.5 |
|  |  |  | extraction protocol | 34 | 2 | 0.001 | 1.0 | 1 | 2.17 | 0.05 | **0.004** |
|  |  |  | storage solution x extraction protocol | not applicable | | | | 1 | 0.86 | 0.02 | 0.7 |
|  |  | RPCA | host subject | 34 | 4 | 1.98 | 0.1 | 3 | 35.03 | 0.76 | **0.001** |
|  |  |  | storage solution | 34 | 2 | 0.46 | 0.5 | 1 | 0.07 | 0.0005 | 0.9 |
|  |  |  | extraction protocol | 34 | 2 | 0.87 | 0.3 | 1 | 4.15 | 0.03 | **0.04** |
|  |  |  | storage solution x extraction protocol | not applicable | | | | 1 | 2.81 | 0.02 | 0.1 |
|  |  | UniFrac | host subject | 34 | 4 | 2.25 | 0.2 | 3 | 4.65 | 0.31 | **0.001** |
|  |  |  | storage solution | 34 | 2 | 0.59 | 0.5 | 1 | 0.96 | 0.02 | 0.5 |
|  |  |  | extraction protocol | 34 | 2 | 0.22 | 0.6 | 1 | 2.18 | 0.05 | **0.004** |
|  |  |  | storage solution x extraction protocol | not applicable | | | | 1 | 0.84 | 0.02 | 0.7 |
|  |  | weighted UniFrac | host subject | 34 | 4 | 0.38 | 0.8 | 3 | 2.03 | 0.17 | 0.07 |
|  |  |  | storage solution | 34 | 2 | 0.40 | 0.6 | 1 | 0.15 | 0.004 | 0.9 |
|  |  |  | extraction protocol | 34 | 2 | 0.14 | 0.7 | 1 | 1.89 | 0.05 | 0.2 |
|  |  |  | storage solution x extraction protocol | not applicable | | | | 1 | 0.10 | 0.003 | 1.0 |
|  |  | phylo-RPCA | host subject | 34 | 4 | 0.14 | 0.9 | 3 | 40.79 | 0.81 | **0.001** |
|  |  |  | storage solution | 34 | 2 | 0.59 | 0.4 | 1 | 0.06 | 0.0004 | 0.9 |
|  |  |  | extraction protocol | 34 | 2 | 1.74 | 0.1 | 1 | 0.28 | 0.002 | 0.7 |
|  |  |  | storage solution x extraction protocol | not applicable | | | | 1 | 2.01 | 0.01 | 0.2 |
|  | surface, floor tile | Jaccard | host subject | 18 | 2 | 0.49 | 0.6 | 1 | 1.71 | 0.09 | **0.001** |
|  |  |  | storage solution | 18 | 2 | 0.68 | 0.5 | 1 | 1.04 | 0.06 | 0.3 |
|  |  |  | extraction protocol | 18 | 2 | 2.33 | 0.2 | 1 | 1.44 | 0.08 | **0.005** |
|  |  |  | storage solution x extraction protocol | not applicable | | | | 1 | 1.03 | 0.06 | 0.3 |
|  |  | RPCA | host subject | 18 | 2 | 0.57 | 0.4 | 1 | 1.46 | 0.07 | 0.3 |
|  |  |  | storage solution | 18 | 2 | 0.03 | 0.8 | 1 | 0.10 | 0.005 | 0.9 |
|  |  |  | extraction protocol | 18 | 2 | 0.004 | 0.9 | 1 | 5.34 | 0.25 | **0.03** |
|  |  |  | storage solution x extraction protocol | not applicable | | | | 1 | 1.64 | 0.08 | 0.2 |
|  |  | UniFrac | host subject | 18 | 2 | 0.22 | 0.7 | 1 | 1.76 | 0.10 | **0.001** |
|  |  |  | storage solution | 18 | 2 | 0.41 | 0.6 | 1 | 1.05 | 0.06 | 0.3 |
|  |  |  | extraction protocol | 18 | 2 | 4.25 | **0.02** | 1 | 1.66 | 0.09 | **0.001** |
|  |  |  | storage solution x extraction protocol | not applicable | | | | 1 | 1.01 | 0.05 | 0.4 |
|  |  | weighted UniFrac | host subject | 18 | 2 | 3.45 | 0.1 | 1 | 13.96 | 0.30 | **0.001** |
|  |  |  | storage solution | 18 | 2 | 4.86 | 0.07 | 1 | 2.57 | 0.06 | **0.04** |
|  |  |  | extraction protocol | 18 | 2 | 2.25 | 0.2 | 1 | 15.62 | 0.34 | **0.001** |
|  |  |  | storage solution x extraction protocol | not applicable | | | | 1 | 1.41 | 0.03 | 0.2 |
|  |  | phylo-RPCA | host subject | 18 | 2 | 0.44 | 0.4 | 1 | 6.29 | 0.20 | **0.008** |
|  |  |  | storage solution | 18 | 2 | 1.52 | 0.2 | 1 | 1.50 | 0.05 | 0.2 |
|  |  |  | extraction protocol | 18 | 2 | 0.01 | 0.9 | 1 | 8.59 | 0.27 | **0.002** |
|  |  |  | storage solution x extraction protocol | not applicable | | | | 1 | 2.58 | 0.08 | 0.09 |
|  | surface, keyboard | Jaccard | host subject | 13 | 2 | 0.30 | 0.7 | 1 | 1.14 | 0.09 | 0.1 |
|  |  |  | storage solution | 13 | 2 | 0.46 | 0.6 | 1 | 1.05 | 0.08 | 0.3 |
|  |  |  | extraction protocol | 13 | 2 | 0.0001 | 1.0 | 1 | 1.23 | 0.10 | **0.05** |
|  |  |  | storage solution x extraction protocol | not applicable | | | | 1 | 1.32 | 0.10 | **0.01** |
|  |  | RPCA | host subject | 13 | 2 | 0.84 | 0.4 | 1 | 0.67 | 0.04 | 0.6 |
|  |  |  | storage solution | 13 | 2 | 0.05 | 0.9 | 1 | 1.11 | 0.06 | 0.3 |
|  |  |  | extraction protocol | 13 | 2 | 2.14 | 0.1 | 1 | 3.56 | 0.20 | **0.04** |
|  |  |  | storage solution x extraction protocol | not applicable | | | | 1 | 4.44 | 0.25 | **0.01** |
|  |  | UniFrac | host subject | 13 | 2 | 0.03 | 0.9 | 1 | 1.16 | 0.09 | 0.1 |
|  |  |  | storage solution | 13 | 2 | 0.17 | 0.7 | 1 | 1.03 | 0.08 | 0.4 |
|  |  |  | extraction protocol | 13 | 2 | 0.28 | 0.7 | 1 | 1.25 | 0.10 | 0.06 |
|  |  |  | storage solution x extraction protocol | not applicable | | | | 1 | 1.28 | 0.10 | **0.04** |
|  |  | weighted UniFrac | host subject | 13 | 2 | 14.75 | **0.001** | 1 | 1.43 | 0.10 | 0.2 |
|  |  |  | storage solution | 13 | 2 | 5.63 | **0.04** | 1 | 1.49 | 0.11 | 0.2 |
|  |  |  | extraction protocol | 13 | 2 | 0.30 | 0.6 | 1 | 2.11 | 0.15 | 0.1 |
|  |  |  | storage solution x extraction protocol | not applicable | | | | 1 | 1.11 | 0.08 | 0.4 |
|  |  | phylo-RPCA | host subject | 13 | 2 | 0.01 | 0.9 | 1 | 0.53 | 0.03 | 0.6 |
|  |  |  | storage solution | 13 | 2 | 0.14 | 0.7 | 1 | 1.31 | 0.07 | 0.3 |
|  |  |  | extraction protocol | 13 | 2 | 2.23 | 0.1 | 1 | 2.49 | 0.14 | **0.1** |
|  |  |  | storage solution x extraction protocol | not applicable | | | | 1 | 5.97 | 0.33 | **0.007** |

**Table S3. Results of dispersion analysis (PERMDISP) and Permutational Multivariate Analysis of Variance (PERMANOVA) for untargeted LC-MS/MS metabolomic data.** For each sample type, the effects of host subject and storage solution (i.e., ethanol vs. isopropanol) are shown for five distance metrics. Singleton features (i.e., those found in only one sample) were excluded. Each test included 999 permutations.

|  |  |  |  | PERMDISP | | | | PERMANOVA | | | |
| --- | --- | --- | --- | --- | --- | --- | --- | --- | --- | --- | --- |
| biomass | sample type | distance metric | factor | *n* | *k* | *F* | *p*-value | *df* | *F* | *R*2 | *p*-value |
| high | stool, human | Jaccard | host subject | 24 | 4 | 1.33 | 0.2 | 3 | 1.91 | 0.22 | **0.001** |
|  |  |  | storage solution | 24 | 2 | 0.42 | 0.6 | 1 | 0.87 | 0.03 | 0.7 |
|  |  | cosine | host subject | 24 | 4 | 4.21 | **0.01** | 3 | 1.98 | 0.22 | **0.004** |
|  |  |  | storage solution | 24 | 2 | 1.44 | 0.2 | 1 | 1.73 | 0.06 | 0.1 |
|  |  | Canberra-Adkins | host subject | 24 | 4 | 0.77 | 0.5 | 3 | 1.78 | 0.21 | **0.001** |
|  |  |  | storage solution | 24 | 2 | 0.04 | 0.8 | 1 | 1.00 | 0.04 | 0.4 |
|  |  | RPCA | host subject | 24 | 4 | 1.01 | 0.3 | 3 | 1.69 | 0.20 | 0.1 |
|  |  |  | storage solution | 24 | 2 | 0.71 | 0.4 | 1 | 1.13 | 0.04 | 0.3 |
|  | stool, mouse | Jaccard | host subject | 24 | 4 | 1.02 | 0.4 | 3 | 1.30 | 0.16 | **0.01** |
|  |  |  | storage solution | 24 | 2 | 0.77 | 0.4 | 1 | 1.63 | 0.07 | 0.0 |
|  |  | cosine | host subject | 24 | 4 | 0.53 | 0.5 | 3 | 1.16 | 0.15 | 0.3 |
|  |  |  | storage solution | 24 | 2 | 1.99 | 0.1 | 1 | 0.98 | 0.04 | 0.5 |
|  |  | Canberra-Adkins | host subject | 24 | 4 | 0.67 | 0.5 | 3 | 1.28 | 0.16 | **0.005** |
|  |  |  | storage solution | 24 | 2 | 1.07 | 0.3 | 1 | 1.65 | 0.07 | 0.0 |
|  |  | RPCA | host subject | 24 | 4 | 0.51 | 0.5 | 3 | 2.30 | 0.26 | **0.05** |
|  |  |  | storage solution | 24 | 2 | 2.74 | 0.09 | 1 | 0.72 | 0.03 | 0.5 |
|  | saliva, before brushing | Jaccard | host subject | 18 | 3 | 0.72 | 0.4 | 2 | 1.23 | 0.14 | 0.08 |
|  |  |  | storage solution | 18 | 2 | 0.01 | 0.9 | 1 | 0.89 | 0.05 | 0.7 |
|  |  | cosine | host subject | 18 | 3 | 1.34 | 0.06 | 2 | 1.38 | 0.15 | 0.2 |
|  |  |  | storage solution | 18 | 2 | 0.16 | 0.6 | 1 | 1.23 | 0.07 | 0.2 |
|  |  | Canberra-Adkins | host subject | 18 | 3 | 0.77 | 0.4 | 2 | 1.47 | 0.16 | **0.002** |
|  |  |  | storage solution | 18 | 2 | 0.06 | 0.8 | 1 | 0.94 | 0.05 | 0.6 |
|  |  | RPCA | host subject | 18 | 3 | 0.64 | 0.4 | 2 | 4.52 | 0.37 | **0.006** |
|  |  |  | storage solution | 18 | 2 | 1.67 | 0.2 | 1 | 1.70 | 0.07 | 0.2 |
|  | saliva, after brushing | Jaccard | host subject | 18 | 3 | 0.47 | 0.6 | 2 | 2.04 | 0.21 | **0.001** |
|  |  |  | storage solution | 18 | 2 | 0.22 | 0.6 | 1 | 1.44 | 0.07 | 0.07 |
|  |  | cosine | host subject | 18 | 3 | 0.61 | 0.3 | 2 | 1.45 | 0.16 | 0.1 |
|  |  |  | storage solution | 18 | 2 | 0.55 | 0.4 | 1 | 0.95 | 0.05 | 0.5 |
|  |  | Canberra-Adkins | host subject | 18 | 3 | 0.75 | 0.5 | 2 | 1.94 | 0.20 | **0.001** |
|  |  |  | storage solution | 18 | 2 | 0.003 | 1.0 | 1 | 1.24 | 0.06 | 0.1 |
|  |  | RPCA | host subject | 18 | 3 | 0.25 | 0.7 | 2 | 4.43 | 0.37 | **0.005** |
|  |  |  | storage solution | 18 | 2 | 8.28 | **0.02** | 1 | 1.05 | 0.04 | 0.4 |
| low | skin, armpit | Jaccard | host subject | 24 | 4 | 0.04 | 1.0 | 3 | 1.86 | 0.19 | **0.01** |
|  |  |  | storage solution | 24 | 2 | 0.16 | 0.6 | 1 | 4.45 | 0.15 | **0.001** |
|  |  | cosine | host subject | 24 | 4 | 0.22 | 0.5 | 3 | 2.35 | 0.22 | **0.01** |
|  |  |  | storage solution | 24 | 2 | 0.16 | 0.5 | 1 | 5.63 | 0.18 | **0.001** |
|  |  | Canberra-Adkins | host subject | 24 | 4 | 0.07 | 0.9 | 3 | 1.61 | 0.18 | **0.01** |
|  |  |  | storage solution | 24 | 2 | 0.29 | 0.5 | 1 | 3.62 | 0.13 | **0.001** |
|  |  | RPCA | host subject | 24 | 4 | 0.41 | 0.5 | 3 | 1.85 | 0.17 | 0.1 |
|  |  |  | storage solution | 24 | 2 | 0.42 | 0.4 | 1 | 8.04 | 0.25 | **0.003** |
|  | skin, hand | Jaccard | host subject | 23 | 4 | 0.12 | 0.8 | 3 | 0.71 | 0.09 | 0.9 |
|  |  |  | storage solution | 23 | 2 | 0.0003 | 1.0 | 1 | 2.37 | 0.11 | **0.01** |
|  |  | cosine | host subject | 23 | 4 | 0.40 | 0.2 | 3 | 1.14 | 0.15 | 0.3 |
|  |  |  | storage solution | 23 | 2 | 0.59 | 0.2 | 1 | 1.79 | 0.08 | 0.1 |
|  |  | Canberra-Adkins | host subject | 23 | 4 | 0.20 | 0.7 | 3 | 0.72 | 0.10 | 0.9 |
|  |  |  | storage solution | 23 | 2 | 0.08 | 0.7 | 1 | 2.28 | 0.10 | **0.01** |
|  |  | RPCA | host subject | 23 | 4 | 0.19 | 0.9 | 3 | 0.30 | 0.04 | 0.9 |
|  |  |  | storage solution | 23 | 2 | 0.94 | 0.3 | 1 | 2.19 | 0.10 | 0.1 |
|  | surface, floor tile | Jaccard | host subject | 12 | 2 | 0.25 | 0.4 | 1 | 0.82 | 0.06 | 0.5 |
|  |  |  | storage solution | 12 | 2 | 1.86 | **0.003** | 1 | 5.10 | 0.34 | **0.003** |
|  |  | cosine | host subject | 12 | 2 | 4.50E-05 | 1.0 | 1 | 0.87 | 0.04 | 0.5 |
|  |  |  | storage solution | 12 | 2 | 103.16 | **0.008** | 1 | 10.41 | 0.51 | **0.001** |
|  |  | Canberra-Adkins | host subject | 12 | 2 | 0.13 | 0.6 | 1 | 0.97 | 0.07 | 0.3 |
|  |  |  | storage solution | 12 | 2 | 1.80 | **0.008** | 1 | 4.76 | 0.32 | **0.001** |
|  |  | RPCA | host subject | 12 | 2 | 0.05 | 0.9 | 1 | 0.22 | 0.01 | 0.8 |
|  |  |  | storage solution | 12 | 2 | 0.06 | 0.9 | 1 | 12.12 | 0.57 | **0.003** |
|  | surface, keyboard | Jaccard | host subject | 11 | 2 | 0.03 | 0.7 | 1 | 1.00 | 0.07 | 0.3 |
|  |  |  | storage solution | 11 | 2 | 0.37 | 0.2 | 1 | 5.49 | 0.38 | **0.003** |
|  |  | cosine | host subject | 11 | 2 | 0.70 | 0.2 | 1 | 1.33 | 0.10 | 0.2 |
|  |  |  | storage solution | 11 | 2 | 0.36 | 0.3 | 1 | 3.91 | 0.30 | **0.02** |
|  |  | Canberra-Adkins | host subject | 11 | 2 | 0.12 | 0.6 | 1 | 1.00 | 0.08 | 0.3 |
|  |  |  | storage solution | 11 | 2 | 0.17 | 0.5 | 1 | 4.11 | 0.31 | **0.004** |
|  |  | RPCA | host subject | 11 | 2 | 0.16 | 0.7 | 1 | 0.72 | 0.04 | 0.5 |
|  |  |  | storage solution | 11 | 2 | 1.42 | **0.04** | 1 | 10.80 | 0.55 | **0.01** |
